# Prognostic and Predicted Significance of FENDRR in Colon and Rectum Adenocarcinoma

**DOI:** 10.3389/fonc.2021.668595

**Published:** 2021-09-21

**Authors:** Fan Yang, Siyu Sun, Fei Yang

**Affiliations:** Department of Gastroenterology, Sheng Jing Hospital of China Medical University, Shenyang, China

**Keywords:** colon and rectum adenocarcinoma, LncRNA, FENDRR, TCGA database, biomarker

## Abstract

**Background:**

The role of fetal-lethal non-coding developmental regulatory RNA (FENDRR) has been explored in various cancers; however, its relationship with colon adenocarcinoma/rectum adenocarcinoma (COAD/READ) remains unclear. The objectives of this study were to identify and assess any associations between FENDRR and COAD/READ using The Cancer Genome Atlas (TCGA) database and the Genetic Data Commons (GDC) Data Portal.

**Methods:**

The records of patients with COAD/READ were collected from the GDC Data Portal. After comparing the expression level of FENDRR in COAD/READ and healthy tissues, we evaluated the association of FENDRR with clinicopathological characters and the survival rate, the impact of FENDRR on prognosis, the biological function of FENDRR, and the relative abundance of tumor-infiltrating immune cells in patients with COAD/READ. Moreover, we aimed to construct a protein-protein interaction (PPI) network for selecting genes and a ceRNA network for presenting mRNA-miRNA-lncRNA interactions.

**Results:**

In patients with COAD/READ, FENDRR expression could differentiate tumor tissues from the adjacent healthy tissues since it was significantly lower in the former than in the latter. High FENDRR expression was correlated with poorer survival and higher tumor stage, current tumor stage, and metastasis stage, and also exhibited high scores for apoptosis, autophagy, and senescence. Immune cell infiltration analysis showed that the high expression group had significantly lower immune and stromal scores. Low FENDRR expression was correlated with poor overall survival (OS), and thus, it could serve as an independent risk factor. The prognostic models constructed in the study performed well for the prediction of OS and disease-specific survival (DFS) using FENDRR expression. Gene set enrichment analysis revealed that vascular smooth muscle contraction, melanogenesis, basal cell carcinoma, and Hedgehog signaling pathways were significantly enriched in patients with high FENDRR expression. Eight hub genes, namely, *PKM*, *ALDOA*, *PFKP*, *ALDOC*, *PYGL*, *CTNNB1*, *PSMA*5, and *WNT5A*, were selected from the PPI network, and a ceRNA network was constructed based on the differentially expressed mRNAs, miRNAs, and lncRNAs to illustrate their regulatory relationships.

**Conclusion:**

FENDRR may serve as a potential biomarker for the diagnosis and prognosis of COAD/READ.

## Introduction

Colon adenocarcinoma/rectum adenocarcinoma (COAD/READ) is the most common pathological type of colorectal cancer (CRC), which ranks third in incidence and second in mortality among all cancers, causing yearly more than 800,000 deaths worldwide (1). CRC primarily affects young individuals (2). The 5-year survival rate of CRC patients with early local tumors is approximately 90%, whereas that of advanced patients is around 14% (3). CRC prognosis and overall mortality rate have improved due to multi-disciplinary treatments; however, the median survival time is18–21 months, and > 50% of patients decease due to recurrence and metastasis (4). Therefore, early diagnosis and effective treatment strategies are necessary for the accurate prediction of prognosis for CRC and especially for COAD/READ. The stage of COAD/READ is the most crucial predictor of prognosis. Nevertheless, the current tumor-node-metastasis (TNM) stage has limited sensitivity in predicting tumor recurrence and metastasis. Besides, imaging examinations are delayed, and serological markers, such as carcinoembryonic antigen (CEA) and carbohydrate antigen 199 (CA-199), have limited predictive value due to low sensitivity and specificity (5). Hence, it is crucial to identify a sensitive and effective COAD/READ marker for accurate diagnosis and prediction of prognosis that may also serve as a new therapeutic target.

Long noncoding RNAs (lncRNAs) are over 200 bp in length with little or no protein encoding capability (6). They are known to play important roles in the occurrence and progression of various diseases and regulate gene expression by interacting with numerous different molecules in tumors. Specifically, several lncRNAs have been identified to trigger the onset of CRC through different pathways (7). Thus, lncRNAs can serve as potential markers for cancer diagnosis and prognosis. Several lncRNAs have been identified to trigger the onset of CRC through different pathways (7).

Fetal-lethal non-coding developmental regulatory RNA (FENDRR) is a newly found lncRNA that has been regarded as a tumor suppressor in various malignancies such as gastric cancer (8), breast cancer (9), and hepatocellular carcinoma (HCC) (10). In HCC, FENDRR is significantly downregulated in tumor tissues and cells, targeting Glypican-3 at the epigenetic level, whereas FENDRR restoration potentially prevents disease progression and metastasis (11); in lung adenocarcinoma, high FENDRR expression is associated with better clinical outcome (12); whereas in renal cell carcinoma, FENDRR downregulation is a poor predictor of prognosis (13). Thus, FENDRR expression level could serve as a prognosis marker; however, information on the relationship between FENDRR and COAD/READ is still limited.

**Figure 1:**
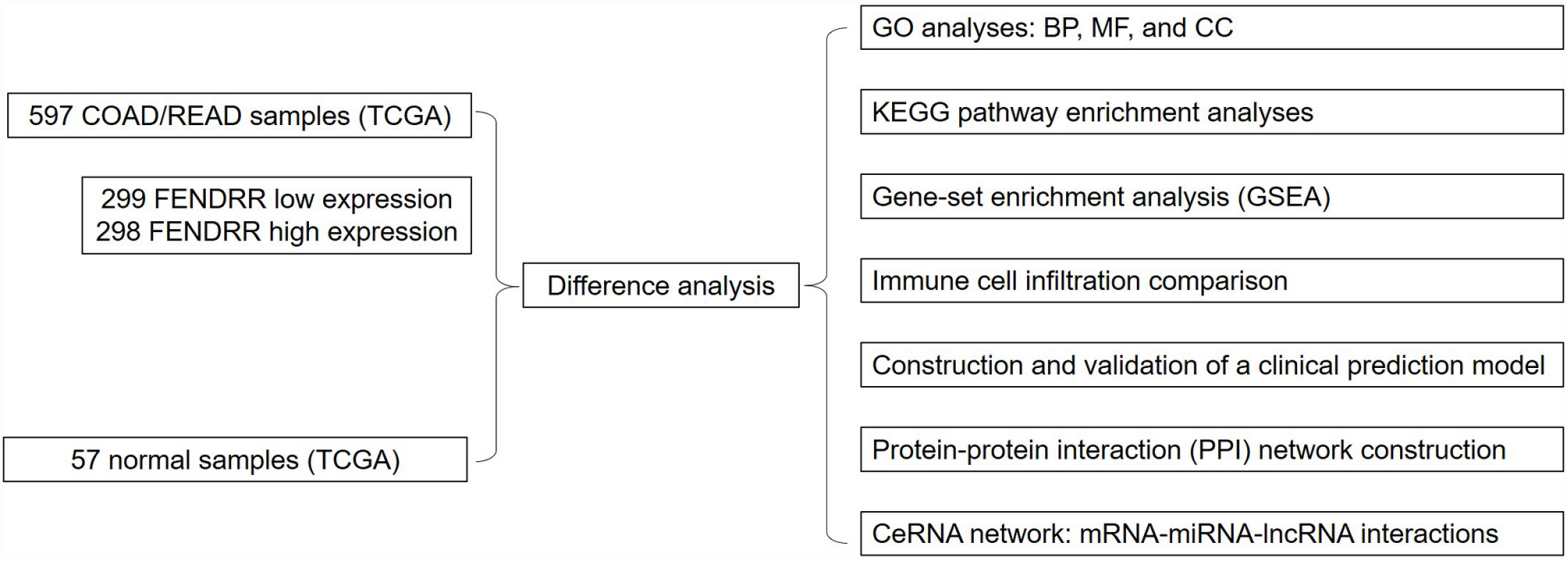
The flowchart of all analysis processes.

This study aimed to evaluate the relationship between FENDRR and COAD/READ using The Cancer Genome Atlas (TCGA) database and the Genetic Data Commons (GDC) Portal. To this end, we obtained RNA-seq data from the GDC Data Portal (https://portal.gdc.cancer.gov/) to assess the prognostic value of FENDRR in COAD/READ and detect any differences in FENDRR expression between COAD/READ tumors and healthy tissues; we analyzed the expression levels of FENDRR and prognostic values based on clinicopathological characteristics of COAD/READ patients to identify any significant associations; we carried out gene-set enrichment analysis to reveal the FENDRR-related biological pathways involved in COAD/READ; and we constructed a competing endogenous RNA (ceRNA) network based on mRNA-miRNA-lncRNA interactions. All analyses carried out in this study are shown in **Figure 1**.

## Materials and Methods

### Data Sources

Gene expression data generated by RNA-sequencing for 607 patients with COAD/READ and 64 healthy tissues were downloaded from the GDC Data Portal (https://portal.gdc.cancer.gov/). The data were classified into mRNA and lncRNA expression profiles. Additionally, clinicopathological characteristics, including gender, age, and tumor stage, as well as prognostic information, were downloaded from the University of California, Santa Cruz Xena website (http://xena.ucsc.edu/). After excluding patients with missing clinical data, 597 tumor samples and 57 healthy tissue samples were included in the study (14). The clinical data for patients with COAD/READ are presented in **Table 1**.

**Table 1:** Baseline characteristics of patients with colon adenocarcinoma/rectum adenocarcinoma (COAD/READ) in the Cancer Genome Atlas (TCGA) database.

Variables	All patients (n = 597)	Low expression (n = 299)	High expression (n = 298)	*p*-value
Gender				0.054
Female	277 (46.4%)	127 (42.5%)	150 (50.3%)	
Male	320 (53.6%)	172 (57.5%)	148 (49.7%)	
Age (years)				0.378
< 60	170 (28.5%)	90 (30.1%)	80 (26.8%)	
≥ 60	427 (71.5%)	209 (69.9%)	218 (73.2%)	
Pathologic stage				0.011*
I	108 (18.1%)	42 (14.0%)	66 (22.1%)	
II	225 (37.7%)	128 (42.8%)	97 (32.6%)	
III	177 (29.6%)	82 (27.5%)	95 (31.9%)	
IV	87 (14.6%)	47 (15.7%)	40 (13.4%)	
T				0.005**
T1	19 (3.2%)	12 (4.0%)	7 (2.3%)	
T2	105 (17.6%)	39 (13.0%)	66 (22.1%)	
T3	408 (68.3%)	207 (69.2%)	201 (67.4%)	
T4	65 (10.9%)	41 (13.8%)	24 (8.2%)	
N				0.547
N0	342 (57.3%)	176 (58.9%)	166 (55.7%)	
N1	145 (24.3%)	73 (24.4%)	72 (24.2%)	
N2	110 (18.4%)	50 (16.7%)	60 (20.1%)	
M				0.089
M0	453 (75.9%)	218 (72.9%)	235 (78.9%)	
M1&MX	144 (24.1%)	81 (27.1%)	63 (21.1%)	

*, **, indicate significance at p < 0.05 and p < 0.01, respectively.

### Differentially Expressed Genes (DEGs)

To analyze the effect of FENDRR, tumor samples from patients with COAD/READ in TCGA were divided into high- and low-expression groups using the median FENDRR level as a threshold. DEGs between the two groups were identified using the package ‘limma’ in r with a log fold change (logFC) > 1.5 and an adjusted *p* < 0.05 as thresholds (15). The results were summarized in a heatmap and volcano plot.

### Function, Pathway, and Gene-Set Enrichment Analyses

Gene Ontology (GO) function and Kyoto Encyclopedia for Genes and Genomes (KEGG) pathway enrichment analyses were carried out using the package ‘clusterProfiler’ in R with a false discovery rate (FDR) of < 0.05 (16). including the sub-ontologies biological process (BP), molecular function (MF), and cellular component (CC), are frequently used for large-scale functional enrichment studies. KEGG is widely used to evaluate enrichment for biological pathways. The R software package clusterProfiler (16) was used for GO annotation and KEGG pathway enrichment analyses of the signature genes. We also performed gene-set enrichment analysis (GSEA) to identify terms enriched in biological processes for DEGs between the high- and low-expression groups using the ‘c2.cp.kegg.v6.2.-symbols’ gene set from the MSigDB database the gene expression profile datasets for patients with COAD/READ. GSEA is a computational method for determining whether a predefined set of genes exhibits significant differences between two biological states. It is typically used to estimate changes in pathways and biological processes in expression datasets (17). with an adjusted *p* < 0.05 (17). Genes in the relevant pathways were further searched against the GeneCard database. A single-sample gene-set enrichment analysis (ssGSEA) was performed to calculate enrichment scores for different pathways in each patient.

### Immune Cell Infiltration and Enrichment Scores

The ssGSEA algorithm was applied to quantify the relative abundance of tumor-infiltrating immune cells (i.e., CD8^+^ T cells, dendritic cells, macrophages, and regulatory T cells) in patients with COAD/READ as described previously (18). Enrichment scores, which represent the level of infiltration by each immune cell type in each sample, were computed using the package ‘gsva’ in R with ssGSEA (17).

Immune activity (i.e., immune cell infiltration level) and stromal scores were calculated for each tumor using the package ‘estimate’ in R (19). Mann-Whitney U tests were used to compare the levels of immune cell infiltration between the high- and low-expression groups.

### Construction and Validation of a Clinical Prediction Model

To further investigate the combined effects of FENDRR expression and clinicopathological characteristics on patient prognosis, univariate and multivariate Cox analyses were used to assess associations with overall survival (OS). Independent prognostic factors were used to construct nomogram models for the prediction of clinical outcomes. Harrell’s concordance index (C-index) was used to quantify the discrimination performance. The actual observed survival rates were used to assess the performance of nomograms, and calibration curves were generated by comparing predicted survival rates.

### Protein-Protein Interaction (PPI) Network Construction and Selection of Hub Genes

A PPI network was constructed using genes from the STRING database with a score of >0.4 and visualized using Cytoscape 3.7.2 (20). The Maximal Clique Centrality (MCC) for each node was computed using the plugin ‘CytoHubba’ in Cytoscape, and genes with the top eight MCC values were considered hub genes (21).

### Construction of a ceRNA Network Based on mRNA-miRNA-lncRNA Interactions

The lncRNA-miRNA interaction data were downloaded from the miRcode database, whereas the miRNA-mRNA interaction data from the miRTarBase, miRDB, and TargetScan databases. Differentially expressed miRNAs and lncRNAs between the high- and low-expression groups were identified using the package ‘limma’ in R with a logFC > 1.5 and an adjusted *p* < 0.05 as thresholds. A correlation analysis was performed to evaluate miRNAs that regulated both lncRNAs and mRNAs, and a ceRNA network was constructed using Cytoscape 3.7.2 (20).

### Statistical Analysis

All data processing and analyses were conducted using R 4.0.2. Independent Student’s t-test was used for pairwise comparisons of normally distributed continuous variables; Mann-Whitney U test (i.e., Wilcoxon rank-sum test) for pairwise comparisons of variables that did not follow a normal distribution; and chi-square or Fisher exact tests for pairwise comparisons of categorical variables. Pearson correlation was used to calculate the coefficients for significant associations between the expression levels of different genes. Survival analysis was carried out using the package ‘survival’ in R, and the differences were evaluated using Kaplan-Meier survival curves. The log-rank test was used to assess the difference in survival time between the high- and low-expression groups. Univariate and multivariate Cox analyses were performed to identify independent prognostic factors. The receiver operating characteristic (ROC) curve was generated using the package ‘pROC’ in R (22). The area under the curve (AUC) was calculated to evaluate the accuracy of the risk score for prognostication. All *p* are two-sided, and significance was set at *p* < 0.05.

## Results

### Correlation Between FENDRR Expression and Clinical Characteristics

**Figure 2:**
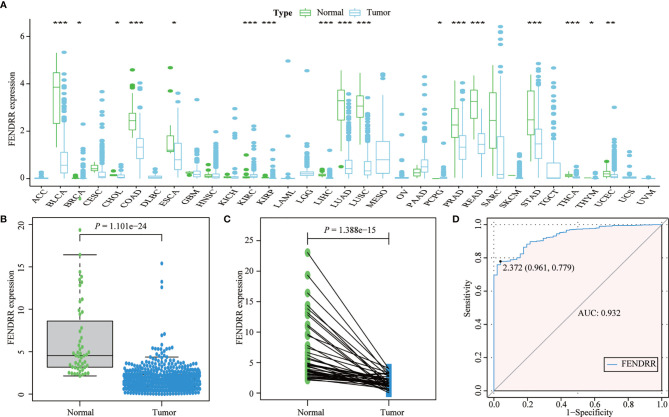
Fetal-lethal non-coding developmental regulatory RNA (FENDRR) expression in patients with colon adenocarcinoma/rectum adenocarcinoma (COAD/READ). **(A)** FENDRR expression in 33 tumor types and adjacent healthy tissues. **(B)** FENDRR expression in tumors and adjacent healthy tissues. **(C)** FENDRR expression in tumors and matched adjacent healthy tissues. **(D)** Receiver operating characteristic (ROC) curve analysis of FENDRR expression in tumors and adjacent healthy tissues. *, **, ***, indicate significance at *p* < 0.05, *p* < 0.01 and *p* < 0.001, respectively.

**Figure 3:**
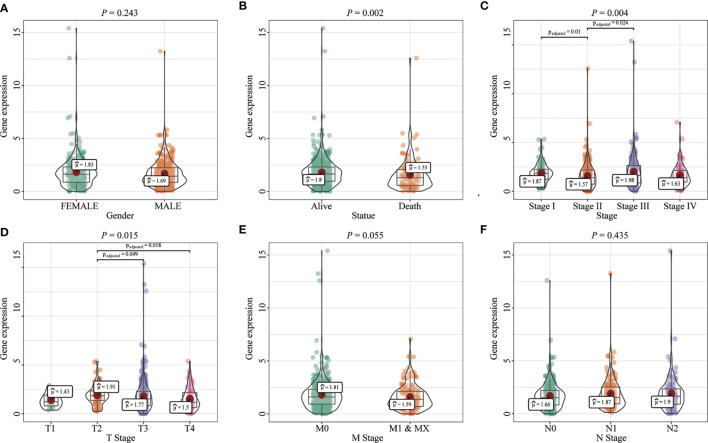
Correlation between fetal-lethal non-coding developmental regulatory RNA (FENDRR) expression and clinicopathological characteristics in patients with colon adenocarcinoma/rectum adenocarcinoma (COAD/READ). **(A–F)** Association of FENDRR expression with poorer survival (*p* = 0.002), higher tumor stage (*p* = 0.004), T stage (*p* = 0.015), M stage (*p* = 0.055), N stage (*p* = 0.435), and (*p* = 0.243).

Most tumors in TCGA exhibited significantly lower FENDRR expression levels than healthy tissues adjacent to the tumor (**Figures 2A–C**). FENDRR expression in tumor tissues was significantly lower than that in adjacent healthy tissues in patients with COAD/READ (*p* < 0.001; **Figure 2B**) or in matched adjacent healthy tissues (*p* < 0.001; **Figure 2C**). A ROC curve analysis showed that FENDRR expression could differentiate tumor tissues from adjacent healthy tissues in patients with COAD/READ (AUC, 0.932; 95% confidence interval, 0.920–0.982; **Figure 2D**). The Kruskal-Wallis and Wilcoxon rank sum tests revealed that high FENDRR expression was closely correlated with poorer survival (*p* = 0.002; **Figure 3A**), higher tumor stage (*p* = 0.004; **Figure 3B**), T stage (*p* = 0.015; **Figure 3C**), and M stage (*p* = 0.055; **Figure 3D**). However, FENDRR expression was not correlated with gender (*p* = 0.243; **Figure 3E**) or N stage (*p* = 0.435; **Figure 3F**).

### Correlation Between FENDRR Expression and Biological Characteristics

**Figure 4:**
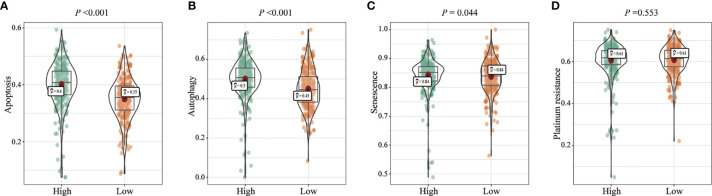
Correlation between fetal-lethal non-coding developmental regulatory RNA (FENDRR) expression and biological pathways. **(A–D)** Association of FENDRR expression with apoptosis (*p* < 0.001), autophagy (*p* < 0.001), senescence (*p* = 0.044), and platinum resistance score (*p* = 0.553).

We analyzed the effects of FENDRR expression on various biological processes in patients with COAD/READ. Patients with high FENDRR expression exhibited higher scores for apoptosis (*p* < 0.001; **Figure 4A**), autophagy (*p* < 0.001; **Figure 4B**), and senescence (*p* = 0.044; **Figure 4C**), whereas FENDRR expression was not associated with the platinum resistance score (*p* = 0.553; **Figure 4D**).

### Correlation Between FENDRR Expression and Immune Cell Infiltration

**Figure 5:**
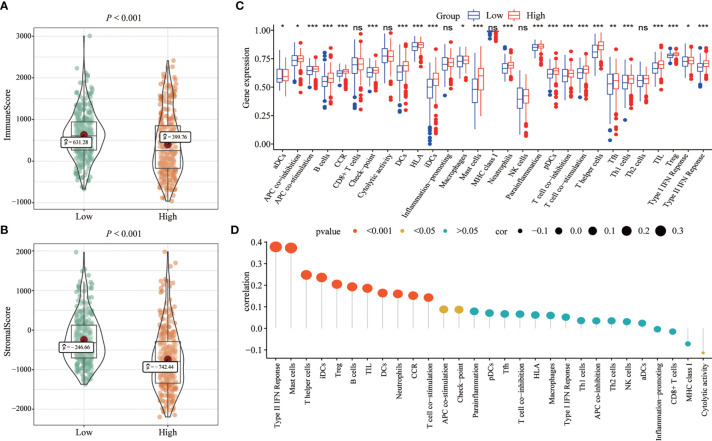
Correlation between fetal-lethal non-coding developmental regulatory RNA (FENDRR) expression and immune cell infiltration. **(A, B)** Association of high FENDRR with immune scores and stromal scores (*p* < 0.001). **(C)** Association of high and low FENDRR expression with the proportions of multiple subtypes of immune cells. **(D)** Lollipop chart of FENDRR expression and different immune cell subtypes. Circle sizes represent the magnitude of the correlation coefficients. Colors represent the significance level (i.e., *p*-values). *, **, ***, indicate significance at *p* < 0.05, *p* < 0.01 and *p* < 0.001, respectively. ns, indicates no statistical significance.

We analyzed the effects of high and low expression levels on immune characteristics and 22 different immune cell subtypes. Patients in the high-expression group exhibited significantly lower immune and stromal scores than those in the low-expression group (*p* < 0.001; **Figures 5A, B**). The levels of infiltration of multiple subtypes of immune cells differed significantly between the high- and low- expression groups (*p* < 0.05, **Figure 5C**). As shown in **Figure 5D**, we found a positive correlation between the expression of FENDRR and type II interferon levels.

### Prognostication Based on FENDRR Expression

**Figure 6:**
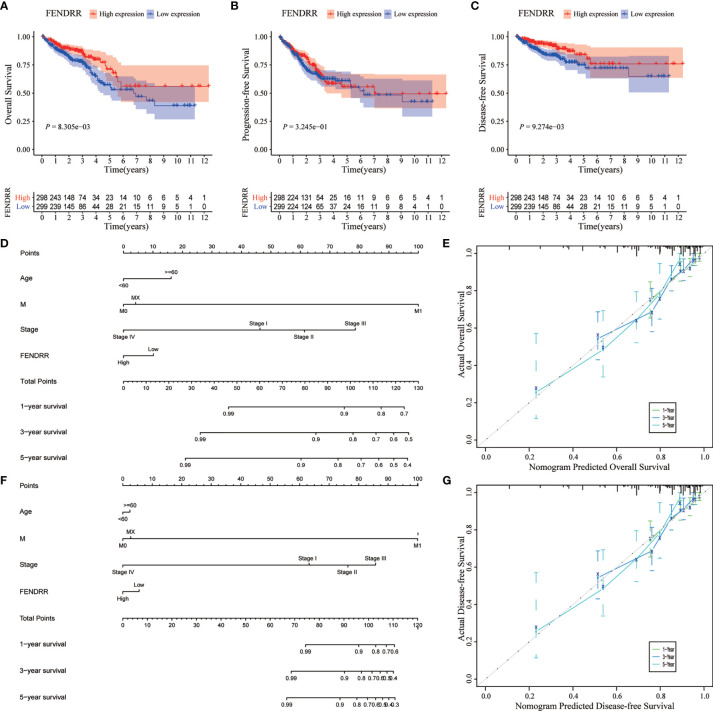
Effect of fetal-lethal non-coding developmental regulatory RNA (FENDRR) expression on colon adenocarcinoma/rectum adenocarcinoma (COAD/READ) prognosis. **(A–C)** Kaplan-Meier analysis for overall survival (OS), disease-free survival (DFS), and progression-free survival (PFS) in relation to FENDRR expression. **(D, F)** Nomograms for predicting OS and DFS. **(E, G)** Calibration curves of nomograms. X-axis, survival status predicted by the nomogram; Y-axis, actual observed survival status.

We analyzed the association between FENDRR expression and prognostic indicators, including OS, progression-free survival (PFS), and disease-specific survival (DFS). The results showed that low FENDRR expression was correlated with poor OS (Log-rank *p* < 0.001; **Figure 6A**) and poor DFS (log-rank *P* < 0.001; **Figure 6C**); however, no significant association was found with PFS (Log-rank *p* = 0.324; **Figure 6B**). Univariate and multivariate Cox regression analyses showed that FENDRR expression level was an independent risk factor for OS (**Table 2**).

**Table 2:** Univariate and multivariate Cox analyses of fetal-lethal non-coding developmental regulatory RNA (FENDRR) expression data for the prediction of overall survival (OS).

Variables	Univariate Cox analysis		Multivariate Cox analysis	
	HR (95% CI)	*P*-value	HR (95% CI)	*P*-value
Age (≥ 60 *vs.* < 60 years)	1.73 (1.09–2.77)	0.020*	2.30 (1.42–3.70)	< 0.001^***^
Gender (male *vs.* female)	1.08 (0.74–1.56)	0.678	0.82 (0.56–1.20)	0.305
T stage (T3 & T4 *vs.* T1 & T2)	3.08 (1.50–6.33)	0.002**	1.93 (0.92–4.03)	0.082
N stage (N1 & N2 *vs.* N0)	2.82 (1.93–4.14)	< 0.001***	0.46 (0.17–1.26)	0.129
M stage (M1 & MX *vs.* M0)	2.86 (1.98–4.15)	< 0.001***	1.94 (1.28–2.93)	0.002**
Stage (III + IV *vs.* I + II)	3.22 (2.18–4.77)	< 0.001***	5.62 (1.91–16.46)	0.002**
FENDRR (high *vs.* low)	0.60 (0.41–0.88)	0.009**	0.59 (0.40–0.88)	0.009**

**, ***, indicate significance at p < 0.01 and p < 0.001, respectively.

We then incorporated the expression status of FENDRR into a multivariate COX analysis to identify significant clinicopathological characteristics (*p* < 0.05) that were used to construct nomograms for predicting OS and DFS in patients with COAD/READ (**Figures 6D, F**). Based on the C-index, the nomograms exhibited excellent discrimination performance (OS, 0.740 [0.690–0.790]; DFS, 0.803 [0.748–0.858]). Furthermore, we tested the predictive power of the models using calibration charts. We compared the nomogram-predicted 1-year, 3-year, and 5-year OS and DFS with the actual patient data and observed a high concordance between the predicted values and observed data. Overall, these results suggested that the constructed prognostic models performed well for the prediction of OS and DFS (**Figures 6E, G**).

### Correlation Between FENDRR Expression and Genome-Wide Expression Profiles

**Figure 7:**
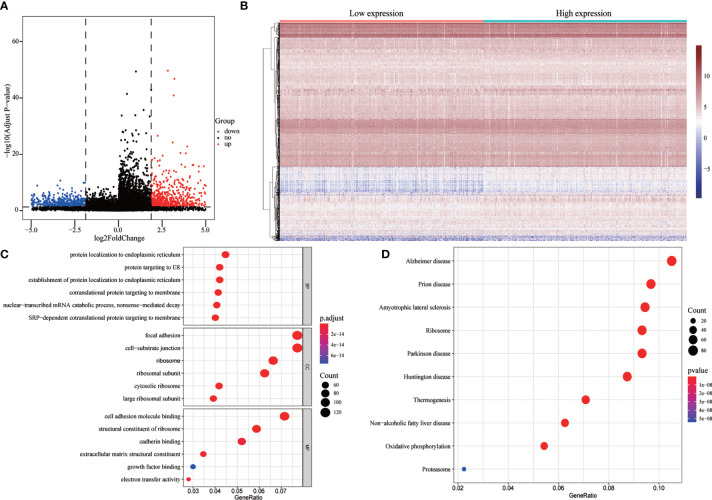
Analysis of differentially expressed genes (DEGs) between high and low fetal-lethal non-coding developmental regulatory RNA (FENDRR) expression groups. **(A, B)** Volcano plot and heat map of DEGs. **(C, D)** DEGs enriched for biological signaling pathways.

**Figure 8:**
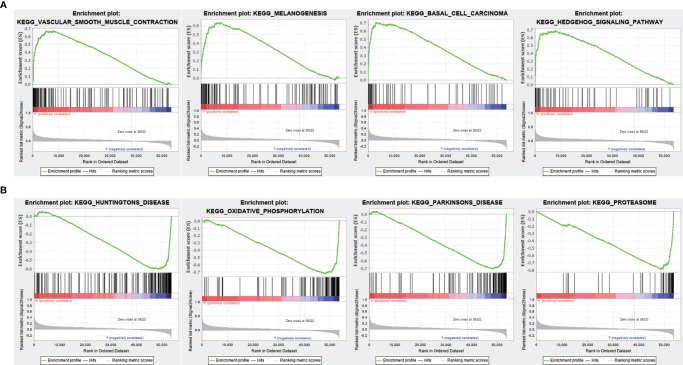
Gene set enrichment analysis (GSEA) based on fetal-lethal non-coding developmental regulatory RNA (FENDRR) expression. **(A)** Association of FENDRR expression with vascular smooth muscle contraction, melanogenesis, and basal cell carcinoma pathways. **(B)** Association of FENDRR expression with Huntington’s disease, oxidative phosphorylation, and other pathways.

To evaluate the correlation between FENDRR expression and genome-wide expression profiles, we divided patients with COAD/READ into high- and low-expression groups based on the median expression level of FENDRR and then analyzed DEGs between the two groups. Using |logFC| > 1.5 and FDR < 0.05 as thresholds, 1,632 DEGs were identified between the low- and high-expression groups (**Figures 7A, B**). GO and KEGG analyses showed that DEGs were enriched in various biological process terms, such as co-translational protein targeting to membrane, signal-recognition particle-dependent co-translational protein targeting to membrane, and ribosomes (**Figures 7C, D**). GSEA showed that various pathways, such as Vascular smooth muscle contraction, melanogenesis, basal cell carcinoma, and Hedgehog signaling, were significantly enriched in patients with high FENDRR expression (**Figure 8A**). Other pathways, including Huntington’s disease, oxidative phosphorylation, Parkinson’s disease, and proteasome pathways, were significantly downregulated in patients with high FENDRR expression (**Figure 8B**).

### PPI and ceRNA Networks

**Figure 9:**
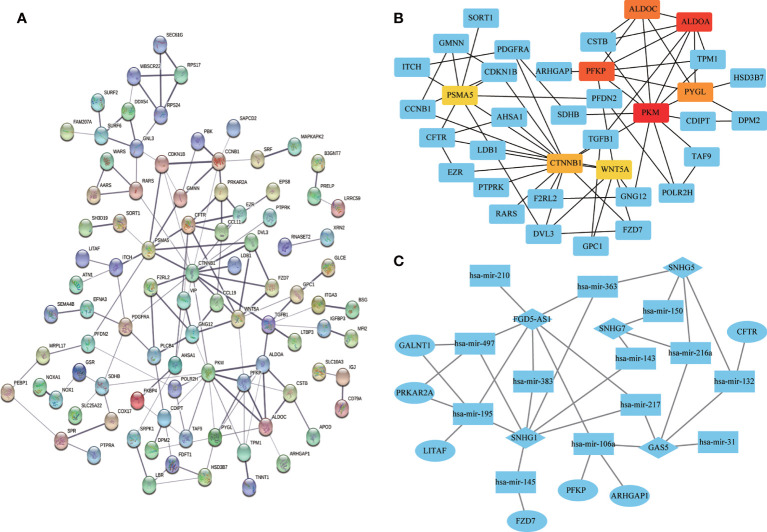
Visualization of protein-protein interaction (PPI) and ceRNA networks. **(A)** PPI network of 136 genes associated with colon adenocarcinoma/rectum adenocarcinoma (COAD/READ). Each node represents a gene. **(B)** Hub genes in the PPI network. Red and yellow nodes represent eight hub genes. **(C)** CeRNA network based on differentially expressed mRNAs, miRNAs, and lncRNAs. Ovals, diamonds, and squares represent mRNAs, lncRNAs, and miRNAs, respectively.

Univariate Cox regression analysis of all DEGs revealed 136 genes significantly associated with prognosis (*p* < 0.05). A PPI network was constructed using the signature genes, and the top eight hub genes (*PKM*, *ALDOA*, *PFKP*, *ALDOC*, *PYGL*, *CTNNB1*, *PSMA*5, and *WNT5A*) were selected using the MCC algorithm (**Figures 9A, B**). A ceRNA network was constructed based on the differentially expressed mRNAs, miRNAs, and lncRNAs to illustrate their regulatory relationships (**Figure 9C**).

## Discussion

COAD/READ accounts for approximately 90% of all CRC, one of the most common cancers worldwide with a high relapse rate (23, 24). Although the implementation of comprehensive treatments, such as surgery and preoperative/postoperative radiotherapy combined with chemotherapy, has markedly improved the 5-year survival rate of CRC patients, invasion and metastasis signify an abominably ominous prognosis (25). Moreover, early diagnosis and the follow-up monitoring of COAD/READ remains a challenge due to the lack of early specific symptoms (26). Colonoscopy is considered the gold standard for detecting and monitoring COAD/READ, but it is an invasive procedure that often causes discomfort and cannot detect metastases early (27, 28). Therefore, it is crucial to find new prognostic and therapeutic targets to improve the clinical strategies and outcomes of COAD/READ.

FENDRR was first reported to be involved in embryonic development as an essential regulatory RNA during embryonic development and differentiation by mediating the epigenetic modification of the target promoter (29). A previous study showed that FENDRR downregulation in mice changes the expression of transcription factors related to the differentiation of the lateral mesoderm, resulting in fatal malformations of the heart and ventral body wall (30). After mutation of FENDRR, the levels of H3K4me3 in the promoter regions of Gate6 and Nkx2.5 increase, affecting cardiac differentiation (29). In gastric cancer, FENDRR suppresses cell invasion, and migration by downregulating fibronectin1, whereas its low expression is associated with poor prognosis (8). In breast cancer, FENDRR inhibits cell proliferation and is associated with a good prognosis (9). However, information on the involvement of FENDRR in the occurrence and development of CRC is limited. Previous studies suggested that FENDRR attenuated CRC progression by repressing SOX4 and that FENDRR levels are negatively correlated with advanced stage and poor clinical outcomes (31); thus, overexpression of FENDRR suppresses the proliferation, migration, and invasion of CRC cells. Nevertheless, the underlying molecular mechanism of FENDRR affecting CRC, especially COAD/READ, was not defined.

Here, we aimed to investigate the expression of FENDRR in COAD/READ tissues and its potential therapeutic and prognostic value. Analyzing high-throughput RNA sequencing data from the TCGA database, we found that FENDRR was significantly downregulated in COAD/READ tissues compared with the paired healthy tissues. The large sample size determined that the probability of overfitting was minimal, leading to accurate conclusions. The results showed that higher expression of FENDRR in COAD/READ tissues was positively correlated with higher tumor stage, T stage, and M stage, suggesting poor survival rate. Moreover, analysis of FENDRR prognosis in COAD/READ indicated that the expression level of FENDRR was an independent risk factor for OS and could serve as a potential prognostic indicator in patients with COAD/READ (8, 9, 13).

TCGA-based analysis indicated that high FENDRR expression promotes autophagy, apoptosis, and senescence in COAD/READ. Furthermore, immune cell infiltration analysis suggested that high FENDRR expression is associated with significantly lower immune and stromal scores and positively correlated with type II interferon levels. FENDRR reportedly promotes apoptosis in hepatic carcinoma, gastric cancer, breast cancer, and even in acute pancreatitis (9, 32–34). In addition to apoptosis, we found that FENDRR was also involved in regulating other biological processes in COAD/READ such as autophagy and senescence; however, the regulatory relationships need to be verified experimentally. A previous study demonstrated that FENDRR enhances IFNγ-induced M1 macrophage polarization, suggesting that FENDRR may be involved in COAD/READ immune regulation (35). Our GSEA results showed that high FENDRR expression was positively correlated with vascular smooth muscle contraction, melanogenesis, basal cell carcinoma, and Hedgehog signaling, but negatively correlated with the proteasome, Huntington’s disease, oxidative phosphorylation, and Parkinson’s disease. Previous studies explored the roles of Hedgehog signaling in the formation, proliferation, and metastasis of CRC and suggested potential treatment, prognosis, and prevention targets (36). The involvement of oxidative phosphorylation (37) and proteasome pathways (38) in CRC progression has been previously reported, but all the other pathways identified in the present study need further exploration.

LncRNAs, as ceRNAs, regulate gene expression by competitively binding microRNAs, which cause gene silencing. In cervical cancer, FENDRR acts as a ceRNA, inhibiting tumor progression by upregulating tubulin alpha1A (TUBA1A) in a miR-15a/b-5p-dependent manner (39). In HCC, FENDRR acts as a sponge of miR-362-5p, promoting apoptosis and deactivating the p38-MAPK pathway (32). In gastric cancer, FENDRR affects cell malignant activity *via* the miR-214-3P/TET2 axis, whereas in prostate cancer, it reduces malignancy by competitively binding miR-18a-5p with Runt-related transcription factor 1 (RUNX1) (33, 40). In CRC, FENDRR increases the expression of growth inhibitor 4 (ING4) by interacting with miR-18a-5p (41). In the present study, we selected the top eight hub genes from the PPI network and constructed a ceRNA network based on the differentially expressed mRNAs, miRNAs, and lncRNAs, highlighting the regulating role of FENDRR in the occurrence and development of COAD/READ.

Our study had some limitations: 1) we collected data from a public database that lack important clinical factors (i.e., treatments received by patients) and were inconsistent since experiments were performed in different laboratories; 2) the number of healthy subjects used as controls was markedly different from that of cancer patients in the current study; 3) as a retrospective study, it failed to failed to include different races and regions that may affect RNA expression in COAD/READ;. Overall, although a multi-center study in public databases intends to complement the short-comings of single center studies, retrospective studies still have their own limits, especially nonuniform intervening measures and absence of some information. Therefore, prospective studies should be performed in the future to avoid analysis bias arising from the retrospective nature of the current study. 4) onlyRNA sequencing data from TCGA were analyzed and, consequently, additional important signaling pathways associated with FENDRR may have been missed. Therefore, further research is necessary to verify the binding affinities between lncRNAs, miRNAs, and mRNAs predicted in the present study as well as to. Thus, there are several areas in which further work is needed to deepen our understanding. First, as the response of COAD/READ, it would be interesting to investigate the basic expression and function of FENDRR in COAD/READ. a clean loss-of-function and gain-on-function study with tissue-type specificity and cell-type specificity remains warranted. A recent series of molecular experiments about other tumors may prove strong evidence for the possible phenotype and pathway regulation of FENDRR regulation underlying COAD/READ. Third, the co-expression and interaction among hub genes is a new exciting frontier that awaits further investigation. Co-Immunoprecipitation and pull-down assays would suggest a powerful evidence for molecular mechanisms in the development of COAD/READ. Finally, further research is needed to confirm the ceRNA network centered by FENDRR and its role in COAD/READ, dual luciferase reporter assay, RIP and RNA pull down would be effective means to solve those problems. Overall, additional studies are needed to improve the statistical power and validate our findings.

In summary, we found that decreased FENDRR was related to COAD/READ and poor prognosis. Thus, it may have important roles in the regulation of COAD/READ *via* different pathways; however, additional experiments are needed to demonstrate the biological impact of FENDRR in COAD/READ and further evaluate the relationship of FENDRR expression with clinical characteristics, COAD/READ stage, and prognosis using more extensive clinical data. Overall, we partially unveiled the roles of FENDRR in COAD/READ, and our results might facilitate the identification of a new diagnostic and prognostic biomarker.

## Data Availability Statement

The original contributions presented in the study are included in the article/supplementary material. Further inquiries can be directed to the corresponding author.

## Author Contributions

FaY designed the study and drafted the manuscript. FaY and FeY collected, analyzed, and interpreted the data. FaY, SS, and FeY revised the manuscript. All authors contributed to the article and approved the submitted version.

## Funding

This study was supported by the China Postdoctoral Science Foundation (Grant No. 2020M670101ZX), Doctoral Scientific Research Foundation of Liaoning Province (Grant No. 2019-BS-276), Science and Technology Program of Shenyang (Grant No. 19-112-4-103), Youth Support Foundation of China Medical University (Grant No. QGZ2018058), Scientific Fund of Shengjing Hospital (Grant No. 201801), and 345 Talent Project of Shengjing Hospital (Grant No. 52-30C).

## Conflict of Interest

The authors declare that the research was conducted in the absence of any commercial or financial relationships that could be construed as a potential conflict of interest.

## Publisher’s Note

All claims expressed in this article are solely those of the authors and do not necessarily represent those of their affiliated organizations, or those of the publisher, the editors and the reviewers. Any product that may be evaluated in this article, or claim that may be made by its manufacturer, is not guaranteed or endorsed by the publisher.
